# Jian-Pi-Yi-Shen decoction inhibits mitochondria-dependent granulosa cell apoptosis in a rat model of POF

**DOI:** 10.18632/aging.204320

**Published:** 2022-10-27

**Authors:** Xiao-Lin Jiang, He Tai, Jin-Song Kuang, Jing-Yi Zhang, Shi-Chao Cui, Yu-Xuan Lu, Shu-Bo Qi, Shi-Yu Zhang, Shun-Min Li, Jian-Ping Chen, Xian-Sheng Meng

**Affiliations:** 1 Department of Nephrology, The Fourth of Affiliated Hospital of Guangzhou University of Traditional Chinese Medicine (Shenzhen Traditional Chinese Medicine Hospital), Guangzhou University of Traditional Chinese Medicine, Shenzhen, China; 2 Key Laboratory of Ministry of Education for Traditional Chinese Medicine Viscera-State Theory and Applications, Liaoning University of Traditional Chinese Medicine, Shenyang, China; 3 College of Pharmacy, Liaoning University of Traditional Chinese Medicine, Dalian, China; 4 Department of Internal Medicine, Liaoning Provincial Corps Hospital of Chinese People’s Armed Police Forces, Shenyang, China; 5 Department of Endocrinology and Metabolism, The Fourth People’s Hospital of Shenyang, Shenyang, China; 6 Department of Pharmacy, General Hospital of Northern Theater Command, Shenyang, China; 7 NHC Key Laboratory of Male Reproduction and Genetics, Guangdong Provincial Reproductive Science Institute (Guangdong Provincial Fertility Hospital), Guangzhou, China; 8 College of Basic Medical Science, Chinese Capital Medical University, Beijing, China

**Keywords:** Jian-Pi-Yi-Shen, premature ovarian failure, mitochondrial dysfunction, granulosa cell, apoptosis

## Abstract

As a widely applied traditional Chinese medicine (TCM), Jian-Pi-Yi-Shen (JPYS) decoction maybe applied in curing premature ovarian failure (POF) besides chronic kidney disease (CKD). *In vivo* experiments, 40 female SD (8-week-old) rats were randomized into four groups, namely, control group (negative control), POF model group, JPYS treatment group, and triptorelin treatment group (positive control). JPYS group was treated with JPYS decoction (oral, 11 g/kg) for 60 days, and the triptorelin group was treated with triptorelin (injection, 1.5 mg/kg) for 10 days before the administration of cyclophosphamide (CTX) (50 mg/kg body weight) to establish POF model. We examined apoptosis, mitochondrial function, and target gene (ASK1/JNK pathway and mitochondrial fusion/fission) expression. *In vitro* experiments, the KGN human granulosa cell line was used. Cells were pretreated with CTX (20, 40, and 60 μg/mL) for 24 h, followed by JPYS-containing serum (2, 4, and 8 %) for 24 h. Thereafter, these cells were employed to assess apoptosis, mitochondrial function, and target gene levels of protein and mRNA. *In vivo*, JPYS alleviated injury and suppressed apoptosis in POF rats. In addition, JPYS improved ovarian function. JPYS inhibit apoptosis of granulosa cells through improving mitochondrial function by activating ASK1/JNK pathway. *In vitro*, JPYS inhibited KGN cell apoptosis through inhibited ASK1/JNK pathway and improved mitochondrial function. The effects of GS-49977 were similar to those of JPYS. During POF, mitochondrial dysfunction occurs in the ovary and leads to granulosa cell apoptosis. JPYS decoction improves mitochondrial function and alleviates apoptosis through ASK1/JNK pathway.

## INTRODUCTION

Premature ovarian failure (POF) is a gynecological disease that associates with many complications, and it has undesirable effects on fertility and quality of life in women. POF incidence rises with age increasing and continues to rise annually [1]. Women with POF show infertility, reproductive organ atrophy, neurological/urogenital system dysfunction, cardiovascular risk, and osteoporosis. POF can be induced by chromosomal abnormalities, Fragile-X premutations, autosomal mutations, iatrogenic injuries, such as that caused during chemotherapy and radiotherapy, enzyme inactivity, autoimmune disorders, and unknown etiologies [2].

With the elevated incidence of cancer in young women, the incidence of chemotherapy-induced POF has also increased. Different chemotherapeutic drugs have different mechanisms of action. As an alkylating agent that cross-links DNA, thereby disrupting the cell cycle, cyclophosphamide (CTX) is highly toxic to the ovaries. CTX up-regulates Bax level and down-regulates Bcl-2 level, which can decrease the MMP, disrupt deliver of Cyt-c, and then affect the production of ROS, thereby causing apoptosis [3, 4].

Few methods are available to preserve the ovarian reserve, such as GnRH-a injection and cryopreservation (oocyte), but these methods are far from perfect in that only 30% of transferred embryos result in clinical pregnancy [5] and only 23 % of transferred embryos result in a live birth [6].

Jian-Pi-Yi-Shen (JPYS) decoction is a mixture of two traditional Chinese medicines, namely, Yu-Ping-Feng-San (YPFS) and Da-Huang-Gan-Cao-Tang (DHGCT). The use of YPFS was recorded in Dan Xi Xin Fa by Dan-xi Zhu, and it was used to complement “Qi”. The use of DHGCT was kept in Jin Gui Yao Lue in Han Dynasty, and it was used to remove excessive fluid or static blood through the bowels. As such, POF can be treated by YPFS to replenish “Qi” and by DHGCT to induce purgation. For the past two decades, JPYS has been clinically prescribed as a basic decoction for curing chronic kidney disease (CKD) [7].

According to the teachings of TCM, oocytes originated from the kidneys, so the pathogenesis of POF is associated with an inadequacy in kidney essence. According to these teachings, JPYS can “resolve stasis and activate blood” and “tonify the kidney and fortify the spleen” [7]. As a Chinese traditional medicine, JPYS is comprised of 8 medicinal herbs in Table 1 [8]. In summary, JPYS decoction can used in curing POF.

**Table 1:** Jian-Pi-Yi-Shen formula ingredients.

**Chinese name**	**Full botanical plant names**	**Family name**	**Identifier No.**	**Medicinal part**	**Dosage (g)**
Huang-Qi	*Astragalus membranaceus* (Fisch.) Bunge.	Leguminosae	ild-32156	Dried radix	30
Bai-Zhu	*Atractylis lancea var. chinensis* (Bunge) Kitam	Compositae	gcc-61586	Dried radix and rhizoma	9
Shan-Yao	*Dioscorea oppositifolia* L.	Dioscoreaceae	kew-240597	Dried radix and rhizoma	30
Rou-Cong-Rong	*Cistanche deserticola* Y.C.Ma	Orobanchaceae	kew-2723155	Dried radix and rhizoma	10
Sha-Ren	*Amomum echinosphaera* K.Schum.	Zingiberaceae	kew-219253	Dried fructus	10
Dan-Shen	***S*** *alvia miltiorrhiza Bunge*	Lamiaceae	kew-183206	Dried radix and rhizoma	15
Da-Huang	*Rheum palmatum* L.	Polygonaceae	kew-2425567	Dried radix and rhizoma	10
Gan-Cao	*Glycyrrhiza uralensis* Fisch.	Leguminosae	ild-32406	Dried radix and rhizoma	6

## MATERIALS AND METHODS

### Chemical reagents

JPYS decoction (Beijing Kang-ren-tang Pharmaceutical Co., Ltd in China) were dissolved in deionized water (60° C) to acquire stock solution (1.1 g/mL). The stock solution was conserved in 4° C refrigerator. CTX, triptorelin, and selonsertib were obtained from Solarbio Life Sciences Co., Ltd. (China).

### Chemical component analysis of JPYS decoction

**Figure 1:**
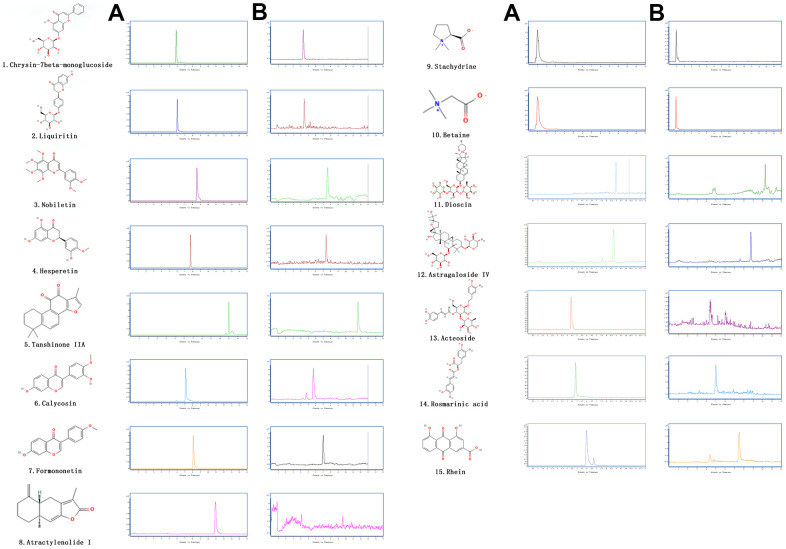
**The extracted ion chromatograms of JPYS decoction.** The extracted ion chromatograms of mixed standards (**A**); The extracted ion chromatograms of JPYS sample: 1.Calycosin-7-O-β-D-glucoside (CAS: 20633-67-4, MRM: 447.01/285.1, retention time: 4.343 min); 2. liquiritin (CAS: 551-15-5, MRM: 419.01/257.2, retention time: 4.463 min); 3. nobiletin CAS: 478-01-3, MRM: 403.01/373.2, retention time: 7.615 min); 4.hesperetin (CAS: 69097-99-0, MRM: 303.01/177.1, retention time: 6.591 min); 5. Tanshinone IIA (CAS: 568-72-9, MRM: 295.01/189.2, retention time: 10.986 min); 6.calycosin (CAS: 20575-57-9, MRM: 285.01/270.1, retention time: 5.767 min); 7. formononetin (CAS: 485-72-3, MRM: 269.01/197.1, retention time: 7.042 min); 8. atractylenolide I (CAS: 73069-13-3, MRM: 231.01/77.2, retention time: 10.185 min); 9. stachydrine (CAS: 471-87-4, MRM: 144.01/58.3, retention time: 1.000 min); 10. betaine (CAS: 478-01-3, MRM: 118.01/58.3, retention time: 1.092 min); 11. dioscin (CAS: 19057-60-4, MRM: 867.99/867.5, retention time: 8.656 min); 12. astragaloside IV (CAS: 96574-01-5, MRM: 783.99/783.5, retention time: 6.442 min); 13. acteoside (CAS: 61276-17-3, MRM: 622.99/161.1, retention time: 4.395 min); 14. rosmarinic acid (CAS: 20283-92-5, MRM: 358.99/161.1, retention time: 4.989 min); 15. rhein (CAS: 478-43-3, MRM: 282.99/239.1, retention time: 7.507 min) (**B**).

JPYS contains eight different medicinal herbs (Table 1). HPLC combined with UPLC-QQQ-MS were used to detect main chemical components of JPYS in previous research, 71 compounds were tentatively identified in JPYS and then using HPLC–QQQ–MS/MS to further qualitative and quantitative 12 compounds [8]. 5 μL injection quantity, 20° C, 0.3 ml/min flow rate) (HPLC) and authenticated with standards. The process was operated in both positive and negative ionization forms (Scan: 100-1500 Da; Fragment: 80-185 V; eV: 4-80 eV). The data were analyzed via SCIEX OS software. Major components of JPYS were validated by real standards. Forms of mass spectrometry and chromatographic are presented in Figure 1.

### Experimental animals

SD rats (body weight, 180–220 g; age, 8 weeks old, 40 female rats and 16 male rats) were used. Rats were housed in cages in room maintained humidity of 45-65 % and a temperature of 20° C, 12-h light/12-h dark (dark at 18:00 h), and administered a pellets and water freely.

40 female rats were randomly divided into 4 groups, namely, control group, POF group, JPYS treatment group, and triptorelin treatment group. For triptorelin group, rats were intramuscularly injected with triptorelin (1.5 mg/kg body weight) on day 1. Triptorelin was dissolved into saline to obtain a 0.75 mg/mL stock solution of. All rats in the other groups were received 0.5 mL saline. Dissolving CTX into saline to obtain stock solutions with 0.5/5 mg/mL. On day 11, all rats, except control group rats, were intraperitoneally injected with CTX (50 mg/kg). For days 12 to 25, all rats, except control group rats, were intraperitoneally injected with CTX (50 mg/kg body weight), whereas control group rats were received saline. With regard to concentration conversion between humans and rats, in terms of the surface area, it was approximately 6.17, and we calculated the dose for intragastric administration. For days 26 to 85, all rats in the JPYS treatment group were intragastrically treated with JPYS (11.0 g/kg body weight), whereas other groups rats were received saline. The treatment protocol is shown in Table 2.

**Table 2:** The treatment schedule details.

**Groups**	**Control**	**Model**	**JPYSP**	**Triptorelin**
**Day**				
1 th day	saline (0.5 ml), i.m.	saline (0.5 ml), i.m.	saline (0.5 ml), i.m.	1.5 mg/kg triptorelin (0.75 mg/ml), i.m.
11 th day	saline (1.0 ml), i.p.	50 mg/kg cyclophosphamide (0.50 mg/ml), i.p.	50 mg/kg cyclophosphamide (0.50 mg/ml), i.p.	50 mg/kg cyclophosphamide (0.50 mg/ml), i.p.
15 th-26 th day	saline (1.0 ml), i.p.	5 mg/kg cyclophosphamide (0.50 mg/ml), i.p.	5 mg/kg cyclophosphamide (0.50 mg/ml), i.p.	5 mg/kg cyclophosphamide (0.50 mg/ml), i.p.
26 th-85 th day	saline (2.0 ml), i.g.	saline (2.0 ml), i.g.	11 g/kg JPYSP (0.55 g/ml), i.g.	saline (2.0 ml), i.g.

**Figure 2:**
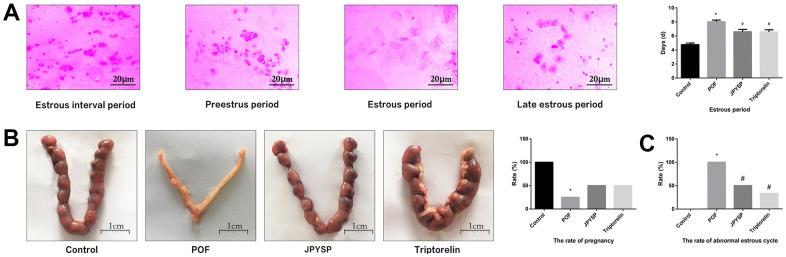
**JPYS improved the abnormal estrous cycle and rate of pregnancy in premature ovarian failure (POF) rats.** Rats were treated with JPYS (11.0 g/kg.d) and pre-treated with triptorelin (1.5 mg/kg) followed by intraperitoneally injected cyclophosphamide (50 mg/kg). (**A**) Estrous cycle of the rats (Estrous interval period: Vagina smear with white blood cells mainly; Preestrus period: With nuclear epithelial cells mainly; Estrous period: With Keratinized epithelial cells mainly; Late estrous period: See Keratinized epithelial cells and white blood cells) (n=6); (**B**) The rate of pregnancy (Female rats of each groups were sent to mate with male rats with ratio of 1:1 for 12 h. The mixture of sperm and vaginal smears seen on the next morning indicated the success of pregnancy, and this was considered as the 0.5 th day of the gestation. The pregnant rats were euthanized on the 13.5 th day of the gestation) (n=4); (**C**) The rate of abnormal estrous cycle (n=6). Data are shown as mean ± SD. *p < 0.05 versus control group, ^#^*p* < 0.05 versus POF group, ^△^*p* < 0.05 versus JPYS group.

To examine the estrous cycle, vaginal smears were obtained from rats in the morning hours (estrous interval period: mostly white blood cells; preestrus period: mostly nuclear epithelial cells; estrous cycle: mostly keratinized epithelial cells; and late estrous cycle: keratinized epithelial cells and white blood cells) (Figure 2A). Approximately 1–5 days following the last irrigation, collecting abdominal aorta blood from all groups rats during the non-estrus period with anesthesia (isoflurane via inhalation anesthesia), followed by oophorectomy. Rats were euthanized. Blood was let to coagulate (1 h) in room temperature, and then serum was acquired through centrifugation. Samples were conserved at -80° C. Ovarian tissues were placed in paraformaldehyde (4%), and some ovarian tissues were frozen by liquid nitrogen and then conserved at -80° C for molecular/protein studies. Lastly, four female rats from each group were mated with male rats with 1:1 ratio for 12 h. A vaginal plug was an indicator of successful pregnancy, and this was considered day 0.5 of gestation. Pregnant rats were euthanized on day 15.5 of gestation.

### Diameter of bilateral ovaries and ovarian index

After anesthesia, the rats were euthanized, and bilateral ovaries were harvested and placed in ice-cold buffer. Fatty tissue surrounding the ovaries was removed, and ovaries were measured the diameter and weighed. The ovarian index was calculated as follows: wet weight of bilateral ovaries (mg)/body weight (g) × 100 % [9].

### Histological assessment of ovarian tissues by HE

Ovarian tissues were fasten by paraformaldehyde (4 %) for 24 h, dehydrated in a graded ethanol series, and then embedded in paraffin. Tissue blocks were cross-sectioned to generate 5-μm-thick sections, and then sections were stained by HE [10]. Sections were observed by Olympus CX33 light microscope and follicle development (primary, secondary, and atretic follicles), as well as the integrity of the corpora lutea were assessed.

### Measurement of serum FSH and E_2_ levels

Arterial blood samples were collected to and levels of FSH and E_2_ were detected using ELISA (Cusabio Biotech Co., Ltd.).

### Morphological evaluation of ovarian tissues by electron microscopy

Ovarian tissues were harvested immediately, cut into smaller pieces of 1 mm^3^, fixed in 4% glutaraldehyde at 4° C for approximately 2 h, and washed with 0.1 M sodium dimethyl arsenate buffer three times. Specimens were placed in osmium tetroxide (1 %) at 4° C for 90 min and washed with distilled water three times. Specimens were dehydrated, and then dehydrated in 100% propionaldehyde two times. The dewatering time of each concentration gradient was 10–15 min. Specimens were embedded in a graded epoxy series (1:3, 1:1, and 3:1) and polymerized at 35° C for 24 h, 45° C for 24 h, and 60° C for 24 h. Tissue blocks were sectioned to generate 70–90 nm-thick sections, which were double stained with lead citrate/uranyl acetate, and then observed with a Hitachi H-7650 electron microscope.

### TUNEL assay

TUNEL was applied to assess cell apoptosis. Five-micron-thick cross-sections were deparaffinized, rehydrated, and treated with protease K (10 μg/mL) for 15 min. Cross-sections were incubated in TUNEL reaction mixture in 37° C for 60 min, then followed by washing. Nuclei were stained with 0.1 μg/mL DAPI, and cross-sections were mounted. Cross-sections were examined under a Canon fluorescent microscope. Eight random fields per sample were examined by investigators, and then number of TUNEL-positive cells was determined.

### Measurement of caspase-3/9 activity

Caspase-3/9 activity of ovarian tissues was measured using related kit. A piece of ovarian tissue (10 mg) was put into the reaction buffer, and then incubated (37° C) for 2 h. A fluorimeter was used to quantify the release of the catalyzed enzyme at an absorbance of 405 nm.

### Preparation of mitochondrial suspension and detection of mitochondrial function *in vivo*

After anesthesia, the rats were killed, and ovaries were harvested, and then placed in PBS. After trimming, approximately 50 mg of ovarian tissues were homogenized in isolation buffer, following centrifugation with 700 × g (10 min). The supernate was gathered, and then centrifuged with 7000 × g (10 min). Discarding the supernate, using isolation buffer to wash mitochondrial pellet, and then sample was centrifuged with 7000 × g (10 min) two times. The purified mitochondrial pellet was resuspended to obtain a protein solution (5 mg/mL). The protein concentration was in the range of 100 μg/mL–1000 μg/mL, then mitochondrial suspension was emploied to measure MMP [11], opening of mPTP [12], ROS production, injuried mtDNA [13], mitochondrial oxygen consumption rate [14], RCR [14], mitochondrial respiratory chain complex enzymes [15], and ATP [15].

### RNA extraction, cDNA synthesis, and real-time PCR

Total RNA was separated with Trizol, then the RNA integrity was assessed by spectrophotometry at a wavelength of 260 nm. For quantitative polymerase chain reactions (PCR), and the reaction volume was 40 μL. Cycling conditions were performed. For semi-quantitative PCR [16], and the reaction system was 20 μL. Cycling conditions were according to following: 95° C for 10 min and 95° C for 10 s, then 40 cycles of 60° C for 15 s, 72° C for 20 s, and 72° C for 10 min. The GAPDH mRNA level was emploied for target gene normalization [17]. Primer sequences are shown in Table 3.

**Table 3:** Sequence of primers for RT-PCR and long PCR.

**Target gene**	**Primer sequence**	**Size (bp)**	**Tm (° C)**
OPA1	Forward: 5’-TGGTTCGAGAGTCGGTTGAA-3’	189	56
	Reverse: 5’- CCTCCCAGTGCTTTGGAGTA -3’		56
Mfn1	Forward: 5’-GGGAAGACCAAATCGACAGA-3’	152	57
	Reverse: 5’-CAAAACAGACAGGCGACAAA-3’		57
Mfn2	Forward: 5’-GAGAGGCGATTTGAGGAGTG-3’	165	58
	Reverse: 5’-CTCTTCCCGCATTTCAAGAC-3’		56
Drp1	Forward: 5’-GCCCGTGGATGATAAAAGTG-3’	215	56
	Reverse: 5’-TGGCGGTCAAGATGTCAATA-3’		56
Fis1	Forward: 5’-AGATGGACTGGTAGGCATGG-3’	84	56
	Reverse: 5’-GACACAGCCAGTCCAATGAG-3’		56
PGC-1α	Forward: 5’-GGACGAATACCGCAGAGAGT-3’	201	59
	Reverse: 5’-CCATCATCCCGCAGATTTAC-3’		56
Tfam	Forward: 5’-TCACCTCAAGGGAAATTGAAG-3’	241	55
	Reverse: 5’-CCCAATCCCAATGACAACTC-3’		56
Long Fragment	Forward:5’-AAAATCCCCGCAAACAATGACCACCC-3’	13400	72
	Reverse: 5’-GGCAATTAAGAGTGGGATGGAGCCAA-3’		72
Shrot Fragment	Forward: 5’-CCTCCCATTCATTATCGCCGCCCTGC-3’	235	60
	Reverse: 5’-GTCTGGGTCTCCTAGTAGGTCTGGGAA-3’		60
Bax	Forward: 5’-GCGATGAACTGGACAACAAC-3’	200	57
	Reverse: 5’-GATCAGCTCGGGCACTTTAG-3’		58
Bcl-2	Forward: 5’-CGAGTGGGATACTGGAGATGA-3’	236	58
	Reverse: 5’- GACGGTAGCGACGAGAGAAG-3’		59
Caspase-3	Forward: 5’-CCCATCACAATCTCACGGTAT-3’	195	57
	Reverse: 5’-GGACGGAAACAGAACGAACA-3’		58
Caspase-9	Forward: 5’-GCCTCTGCTTTGTCATGGAG-3’	181	56
	Reverse: 5’-AGCATGAGGTTCTCCAGCTT-3’		56
ASK1	Forward: 5’-ACAATGAGCAGACGATTGGC-3’	168	56
	Reverse: 5’-CAGCAAGCCTCTTGGATGTC-3’		56
JNK	Forward: 5’-TGGATTTGGAGGAGCGAACT-3’	69	56
	Reverse: 5’-TCACTGCTGCACCTAAAGGA-3’		56
Cyc-c	Forward: 5’-GGACAGCCCCGATTTAAGTA-3’	121	57
	Forward: 5’-TCAATAGGTTTGAGGCGACAC-3’		58
GAPDH	Forward: 5’- AGGTCGGTGTGAACGGATTTG -3’	20	58
	Reverse: 5’- GGGGTCGTTGATGGCAACA-3’		58

### Total protein isolation and Western blotting

Total proteins were extracted, and then protein level was detected with BCA. Equivalent quantity of total protein were subjected to 8–12%. Following blocking membranes with skim milk, they were incubated separately overnight (Table 4). Immunoreactive proteins were observed with chemiluminescence kit and analyzed with software.

**Table 4:** Antibodies used in the study.

**Antibodies**	**Manufacturer**	**Catalogue No.**	**Observed MW**	**Dilution**
Anti-ASK1	Proteintech	67072-1-1g	110 KDa	1:2000
Anti-p-ASK1	Proteintech	28846-1-AP	120 KDa	1:1000
Anti-JNK	Proteintech	10176-2-AP	46KDa	1:2000
Anti-p-JNK	Proteintech	80024-1-RR	46 KDa	1:2000
Anti-Bcl-2	Proteintech	26593-1-AP	26 KDa	1:1000
Anti-Bax	Proteintech	50599-2-1g	26 KDa	1:6000
Anti-Caspase-3	Proteintech	19677-1-AP	32 KDa	1:1000
Anti-Caspase-9	Proteintech	10380-1-AP	47 KDa	1: 500
Anti-Cyt-c	Proteintech	12245-1-AP	13 KDa	1:3000
Anti-OPA1	Proteintech	66583-1-Ig	100 KDa	1:1000
Anti-Mfn1	Proteintech	13798-1-AP	86 KDa	1:500
Anti-Mfn2	Proteintech	12186-1-AP	86 KDa	1:3000
Anti-Drp1	Proteintech	10656-1-AP	27 KDa	1:1000
Anti-Fis1	Proteintech	66635-1-Ig	15 KDa	1:3000
Anti-PGC1a	Proteintech	66369-1-1g	100 KDa	1:5000
Anti-GAPDH	Proteintech	60004-1-1g	36 KDa	1:20000

### Preparation and compound analysis of JPYS-containing serum

Female SD rats were intragastrically treated with saline or JPYS (11.0 g/kg/d) for 7 days. 2 hours after the last treatment, then the blood was extracted from the aorta ventralis, reserved in 4° C (1 h), and then centrifuged (2000 rpm/min) for 30 min. Serum samples were inactivated in a water bath (56° C) for 30 min.

### Cell culture and establishment of POF model

The KGN human granulosa cell line was purchased from Fenghui Bio-Company (cat. no. CL0544, Changsha, China). Cells (2 × 10^6^ cells) were plated in 100-mm culture dishes and cultured in medium with incubator at 37° C with humidified air containing 5% CO_2_. Medium was replaced every other day. Cells were treated with CTX with different concentrations (20, 40, and 60 μg/mL) and/or JPYS-containing serum at different concentrations (2, 4, and 8 %).

### Assignment of cell groups

Cells were divided into 4 groups including control group, CTX (20, 40, and 60 μg/mL) -induced POF model groups, POF + JPYS (JPYS-containing serum, 2, 4, and 8 %) treatment groups, and POF + GS-49977 (5 μM) treatment group and used for *in vitro* experiments. Then we detect the cell viability through CCK-8.

### Hoechst staining

Cells were fixed in paraformaldehyde (4 %) for 10 min, followed by washing with PBS and stained by Hoechst 33258 for 5 min. Images were observed with fuorescent microscope with an 350 nm excitation wavelength and an 460 nm emission wavelength.

### Assessment of apoptosis with flow cytometry

Cells (1 × 10^5^ cells/mL) were seeded and incubated overnight, and then cells were intervened with JPYS (2, 4, and 8 %) and GS-49977 (5 μM) for 24 h, followed by CTX with different levels (20, 40, and 60 μg/mL) for 24 h. Cells were analyzed via flow cytometry.

### Detection of release of ROS by flow cytometry

Using flow cytometry was emploied to measure ROS levels of different groups. Cells in every group were intervened, collected and suspended by DCFH-DA (10 μmol/L) with a final cell (1 × 10^6^ cells/mL), then cells were incubated for 30 min in 37° C with CO_2_ (5 %), and blended every 5 min. The flow cytometer was used to measure the fluorescence intensity.

### Electron microscopy

Cells were fixed with glutaraldehyde (4 %) in 4° C (2 h), and then washed with 0.1 M sodium dimethyl arsenate, and then centrifuged between the washing steps. The remaining steps was similar to *in vivo* study.

### Preparation of mitochondrial suspension and assessment of mitochondrial function *in vitro*

Mitochondria were seperated via related kit and reserved on ice for using. Mitochondria were resuspended in buffer. KGN cells (1 × 10^5^ cells/mL) were plated in 6-well plates, and then centrifuged (600 × g) for 3–4 min. Throwing away supernate. Finally, cells were stained by JC-1 staining buffer, and then supernate was throwed away. Detecting MMP and mPTP [18, 11].

### Statistical analysis

Statistical analysis was carried out by SPSS 17.0 Software. ANOVA was emploied to compare 4/5 independent groups. Two-to-two comparison among groups was emploied to analyze variance. The LSD-t test was emploied to compare multiple comparisons between 4/5 groups. We defineded *P* < 0.05 represent having statistically significance.

### Data availability

The datasets used and/or analyzed during the current study are available from the corresponding author on reasonable request.

## RESULTS

### Chemical components of JPYS decoction

The chemical components of JPYS were measured by HPLC-Q-TOF-MS/MS. The 15 chemical components including: Calycosin-7-O-β-D-glucoside (contents: 12.58 μg/g; CAS: 20633-67-4), liquiritin (contents: 64.56 μg/g; CAS: 551-15-5), nobiletin (contents: 1.41 μg/g; CAS: 478-01-3), hesperetin (contents: 1.04 μg/g; CAS: 69097-99-0), Tanshinone IIA (contents: 18.40 μg/g; CAS: 568-72-9), calycosin (contents: 6.64 μg/g; CAS: 20575-57-9), formononetin (contents: 7.26 μg/g; CAS: 485-72-3), atractylenolide I (contents: 0.40 μg/g; CAS: 73069-13-3), stachydrine (contents: 0.90 μg/g; CAS: 471-87-4), betaine (contents: 17.79 μg/g; CAS: 478-01-3), dioscin (contents: 9.82 μg/g; CAS: 19057-60-4), astragaloside IV (contents: 28.88 μg/g; CAS: 96574-01-5), acteoside (contents: 24.58 μg/g; CAS: 61276-17-3), rosmarinic acid (contents: 28.86 μg/g; CAS: 20283-92-5), and rhein (contents: 6.17 μg/g; CAS: 478-43-3). The extracted-ion chromatograms are described in Figure 1A, and ESI-Q-TOF MS/MS spectra of the five components are described in Figure 1B. 2D structure of chemical components were seeked in https://pubchem.ncbi.nlm.nih.gov/.

### JPYS improved the estrous cycle length, pregnancy rate, and abnormal estrous cycle rate in POF rats

To evaluate degree of ovarian injury and protective effects of JPYS, we examined the estrous cycle length (Figure 2A), rate of pregnancy (Figure 2B), and abnormal estrous cycle rate (Figure 2C). The estrous cycle length increased in POF group (*p* < 0.05) but reduced by treatment with JPYS and pretreatment with triptorelin (*p* < 0.05) (Figure 2A). The pregnancy rate decreased in POF group (*p* < 0.05) but increased after JPYS treatment and triptorelin pretreatment (*p* < 0.05) (Figure 2B). The abnormal estrous cycle rate increased in the POF group (*p* < 0.05) but decreased after JPYS treatment and triptorelin pretreatment (*p* < 0.05) (Figure 2C).

### JPYS improved the ovarian index, diameter of bilateral ovaries, ovarian function, and follicle development in POF rats

**Figure 3:**
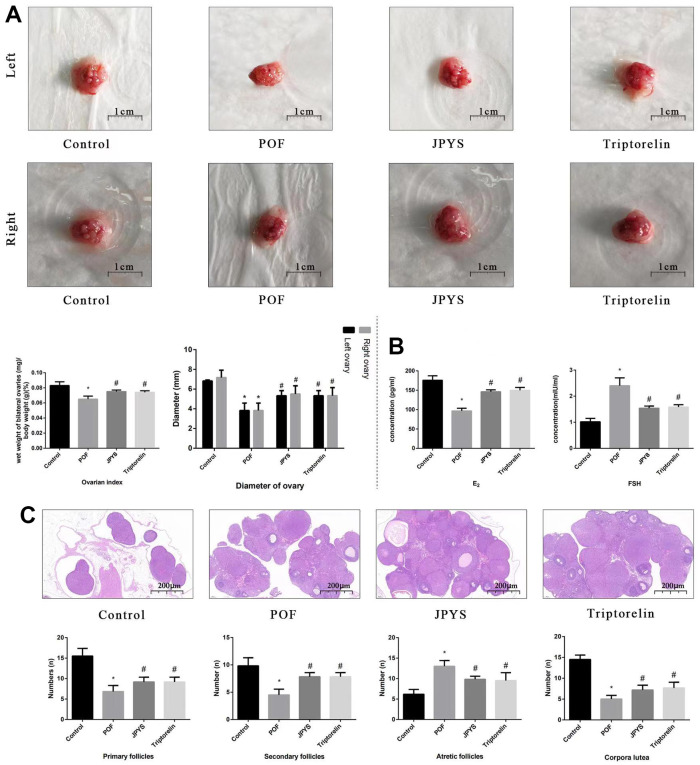
**JPYS improved the ovarian function in premature ovarian failure (POF) rats.** Rats were treated with JPYS (11.0 g/kg.d) and pre-treated with triptorelin (1.5 mg/kg) followed by intraperitoneally injected cyclophosphamide (50 mg/kg). (**A**) Diameter of bilateral ovaries and Ovarian index (Ovarian index= wet weight of bilateral ovaries (mg)/ body weight (g)×100 %, the size of the ovary in POF group were significantly reduced compared with control group), the scale bars represents a length of 1 cm on histology; (**B**) Ovarian function (follicle stimulating hormone (FSH) and oestradiol (E2)); (**C**) Histological assessment of the ovarian tissue using hematoxylin-eosin (HE) staining (All phases of follicles (primary follicles, secondary follicles, and atretic follicles) and corpora lutea were counted), the scale bars represents a length of 20 μm on histology. Data are shown as mean ± SD. *p < 0.05 versus control group, ^#^*p* < 0.05 versus POF group, ^△^*p* < 0.05 versus JPYS group. (n=6).

To further evaluate degree of ovarian injury and protective effects of JPYS, we examined the diameter of bilateral ovaries and ovarian index (Figure 3A), ovarian function (Figure 3B), and follicle development (Figure 3C). The diameter of bilateral ovaries and ovarian index decreased in the POF group (*p* < 0.05) but increased by treatment with JPYS and pretreatment with triptorelin (*p* < 0.05) (Figure 3A). Ovarian function decreased (FSH increased; E_2_ decreased) in the POF group (*p* < 0.05) but increased (FSH decreased; E_2_ increased) after JPYS treatment and triptorelin pretreatment (*p* < 0.05) (Figure 3B). The number of primary and secondary follicles, as well as the surface area of the corpora lutea, decreased in POF group (*p* < 0.05) but increased after JPYS treatment and triptorelin pretreatment (*p* < 0.05). The number of atretic follicles increased in POF group (*p* < 0.05) but reduced following JPYS treatment and triptorelin pretreatment (*p* < 0.05) (Figure 3C).

### JPYS alleviated mitochondrial damage in POF rats

**Figure 4:**
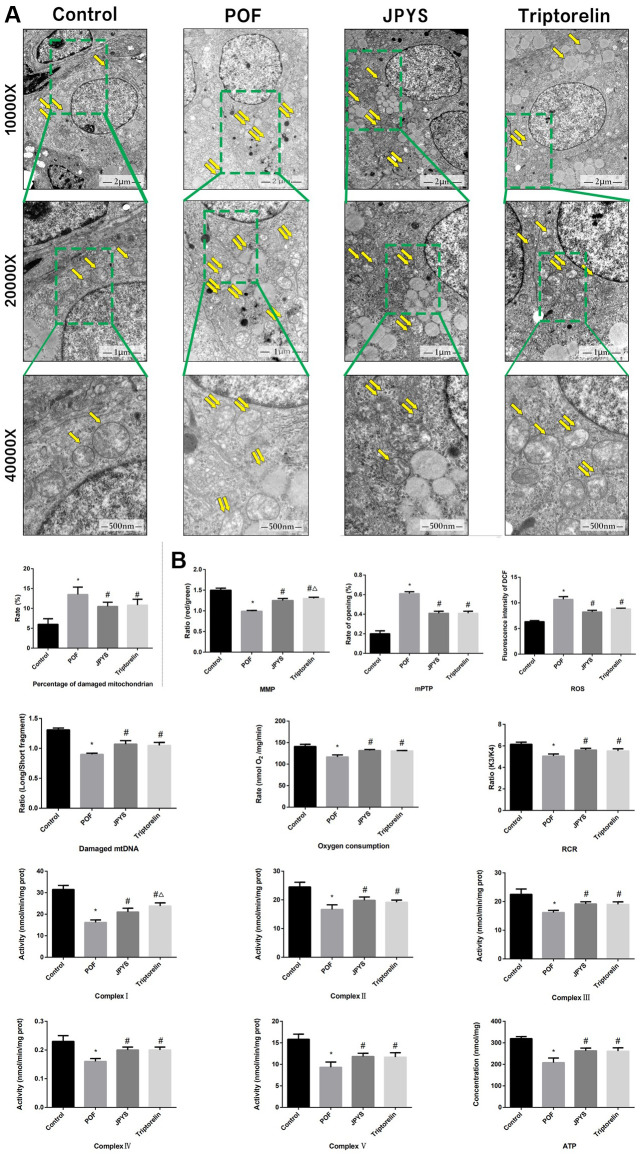
**JPYS improved mitochondrial function in premature ovarian failure (POF) rats.** Rats were treated with JPYS (11.0 g/kg.d) and pre-treated with triptorelin (1.5 mg/kg) followed by intraperitoneally injected cyclophosphamide (50 mg/kg). (**A**) Electron microscope pictures (10,000×; 20,000×; 40 000×) of ovary in POF rats, the scale bars represents a length of 2 μm, 1 μm, and 500 nm on histology respectively. Abnormal mitochondrial (paired yellow arrow) morphology show that mitochondrial membrane rupture or swellings, normal mitochondrial (single yellow arrow) morphology type show that mitochondrial membrane smooth and inner carinulae distinct and percentage of damaged mitochondria; (**B**) The MMP (ratio of red/green), the opening of mPTP (%), the mitochondrial ROS, the mtDNA damage (ratio of long/short fragments), the mitochondrial RCR, mitochondrial oxygen consumption rate, the mitochondrial respiratory chain complex enzymes (I, II, III, IV, and V), and ATP were recorded above. Data are shown as mean ± SD. *p < 0.05 versus control group, ^#^*p* < 0.05 versus POF group, ^△^*p* < 0.05 versus JPYS group. (n=6).

To assess mitochondrial function and examine the protective effects of JPYS, electron microscopy was used (Figure 4). Compared with the ovarian tissues of the control group, which showed normal mitochondria, the results of electron microscopy revealed that ovarian tissues of the POF group showed abnormal mitochondria, with membrane swelling and rupture. The percentage of damaged mitochondria in the POF group was higher than that of mitochondria in the control group (*p* < 0.05). JPYS treatment and triptorelin pretreatment decreased the percentage of damaged mitochondria (Figure 4A). Next, we measured MMP, mPTP opening (%), ROS production, damaged mtDNA, the mitochondrial oxygen consumption rate, RCR, ATP, and mitochondrial respiratory chain complex enzyme in isolated mitochondria. Compared to the control group, ROS production and mPTP opening (%) increased in the POF group (*p* < 0.05). However, ROS production decreased by JPYS treatment and triptorelin pretreatment (*p* < 0.05). Compared to the control group, the mitochondrial oxygen consumption rate, RCR, and the MMP decreased in the POF group (*p* < 0.05). However, JPYS treatment and triptorelin pretreatment increased these indices (*p* < 0.05). Real-time qPCR was used to measure the degree of damaged mtDNA. Compared to the control group, ratio of long/short fragments decreased in the POF group (*p* < 0.05), whereas JPYS treatment and triptorelin pretreatment increased the ratio (*p* < 0.05). Furthermore, the activity of mitochondrial respiratory chain complex enzymes and ATP level were decreased in POF group compared with control group (*p* < 0.05), whereas JPYS treatment and triptorelin pretreatment increased these indices (*p* < 0.05) (Figure 4B).

### JPYS affected mitochondrial biogenesis and ovarian function in POF rats

**Figure 5:**
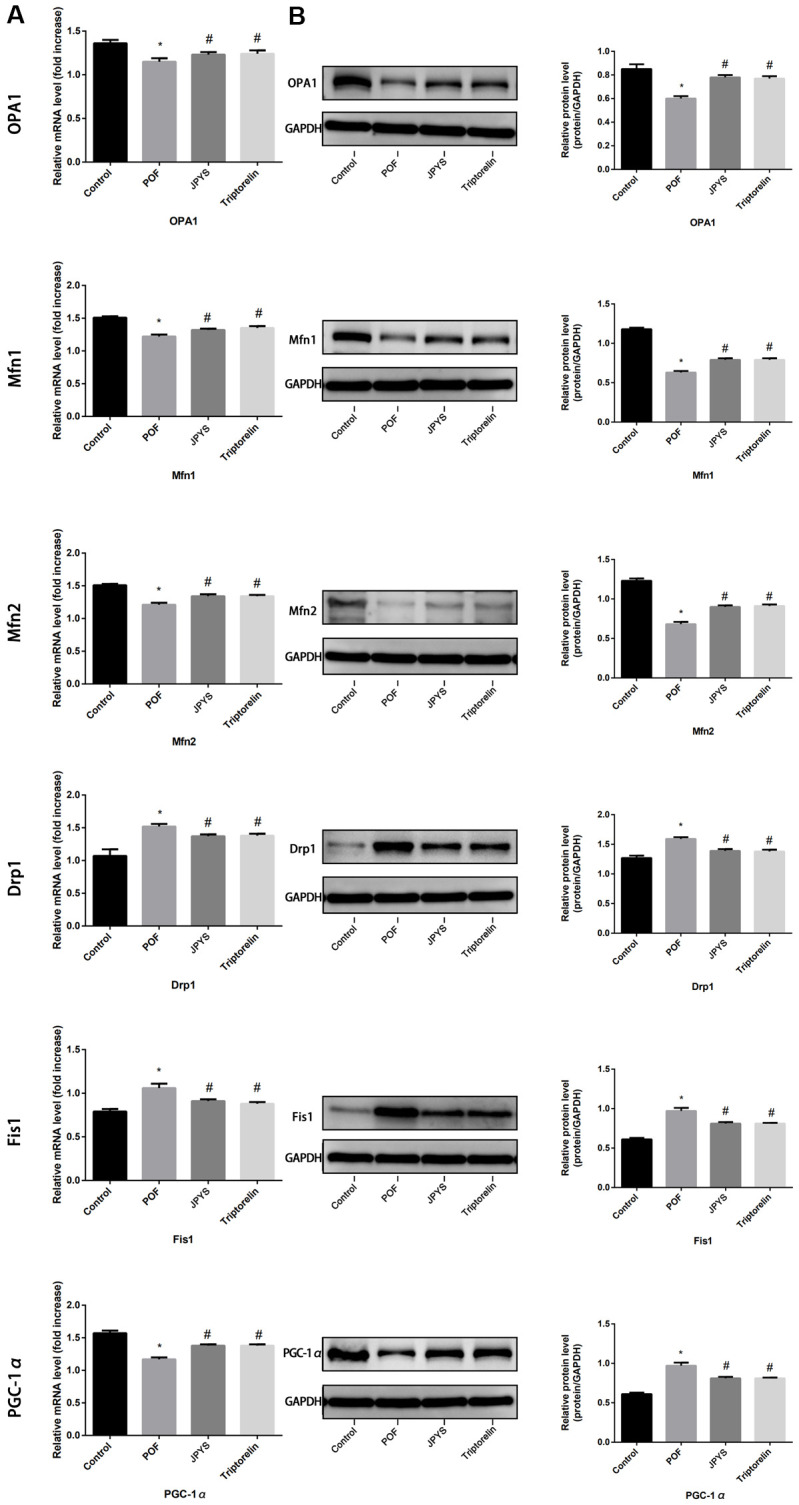
**JPYS improved mitochondrial biogenesis and dynamics in premature ovarian failure (POF) rats.** Rats were treated with JPYS (11.0 g/kg.d) and pre-treated with triptorelin (1.5 mg/kg) followed by intraperitoneally injected cyclophosphamide (50 mg/kg). We used real-time qPCR and western blot to detect mitochondrial biogenesis and dynamics. We chose OPA1, Mfn1, and Mfn2 to represent mitochondrial biogenesis function, and PGC-1α to represent the dynamic mitochondrial fusion, and Drp1 and Fis1 to represent mitochondrial fission. The expression of OPA1, Mfn1, Mfn2, PGC-1α, Drp1, and Fis1 in mRNA (**A**) and protein (**B**) levels. Data are shown as mean ± SD. *p < 0.05 versus control group, ^#^*p* < 0.05 versus POF group, ^△^*p* < 0.05 versus JPYS group. (n=6).

Real-time PCR and Western blotting were used to examine different aspects of mitochondrial function. OPA1, Mfn1, and Mfn2 were selected as markers of mitochondrial biogenesis, PGC-1α was selected as a marker of mitochondrial fusion, and Drp1 and Fis1 were selected as markers of mitochondrial fission. Compared to control group, OPA1, Mfn1, and Mfn2 levels decreased in POF group (*p* < 0.05), whereas JPYS treatment and triptorelin pretreatment increased their mRNA/protein levels (*p* < 0.05). Compared to control group, Drp1 and Fis1 levels increased in POF group (*p* < 0.05), whereas JPYS treatment and triptorelin pretreatment decreased mRNA/protein levels (*p* < 0.05). Compared to the control, PGC-1α levels decreased in POF group (*p* < 0.05), whereas JPYS treatment and triptorelin pretreatment their mRNA/protein levels (*p* < 0.05) (Figure 5).

### JPYS modulated the ASK1/JNK pathway *in vivo*

**Figure 6:**
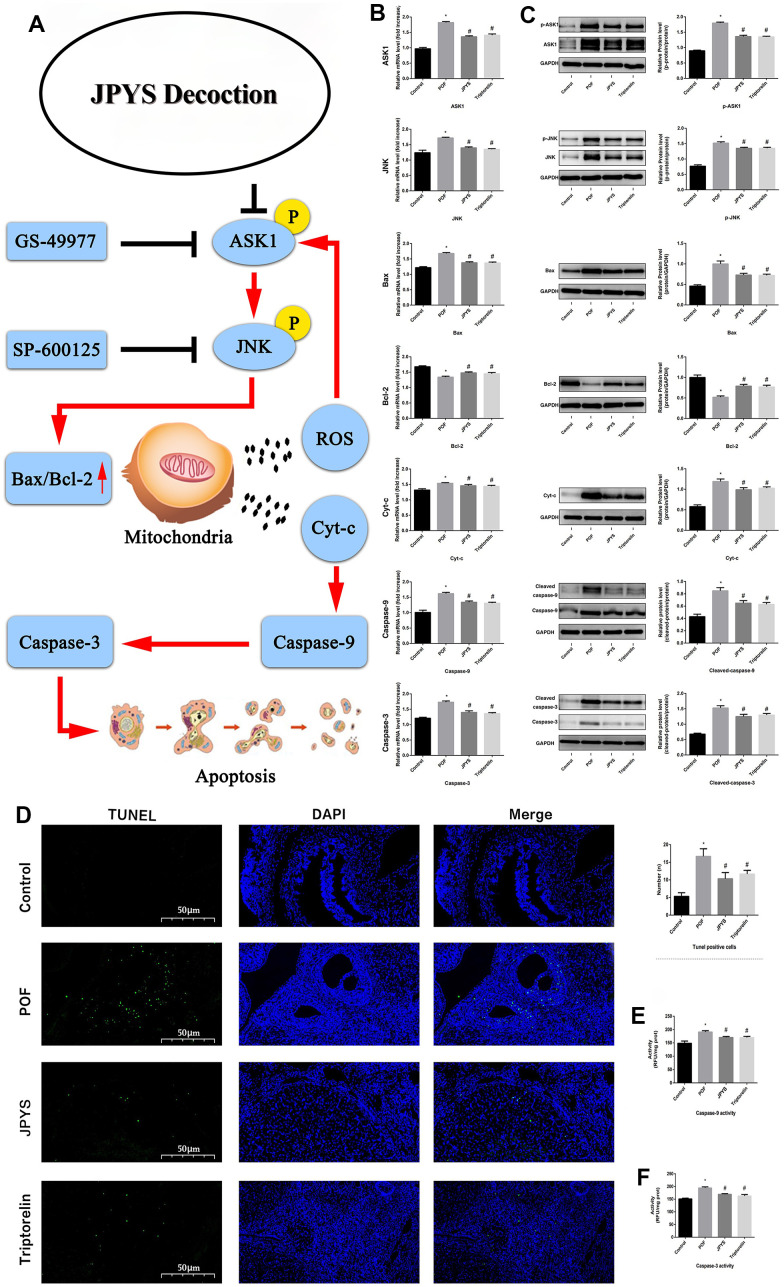
**JPYS improved mitochondrial function and inhibited apoptosis via inhibiting ASK1/JNK pathway.** Rats were treated with JPYS (11.0 g/kg.d) and pre-treated with triptorelin (1.5 mg/kg) followed by intraperitoneally injected cyclophosphamide (50 mg/kg). Graphical abstract (**A**): Mitochondrial dysfunction of the ovarian GC occurs following POF and can lead to apoptosis. JPYS can relieve GC apoptosis by improving mitochondrial function via inhibiting the ASK1/JNK pathway *in vivo* and *vitro*. We used real-time qPCR and western blot to detect the target genes of ASK1/JNK pathway in mRNA (**B**) and protein (**C**) levels. TUNEL positive cells (**D**), the scale bars represents a length of 50 μm on histology. The activity of caspase-9 (**E**). The activity of caspase-3 (**F**). Data are shown as mean ± SD. *p < 0.05 versus control group, ^#^*p* < 0.05 versus POF group, ^△^*p* < 0.05 versus JPYS group. (n=6).

Real-time qPCR and western blotting were used to examine the levels of target genes/proteins within the ASK1/JNK pathway. Compared to the control group, caspase-3/9, Bax, ASK1, JNK, and Cyt-c expression increased (*p* < 0.05) in the POF group. However, the mRNA level of these genes decreased after JPYS treatment and triptorelin pretreatment (*p* < 0.05). Compared to the control, Bcl-2 expression decreased in the POF group (*p* < 0.05). However, the mRNA level of Bcl-2 increased after JPYS treatment and triptorelin pretreatment (*p* < 0.05). JPYS treatment and triptorelin pretreatment inhibited ASK1 and JNK phosphorylation (decreased p-ASK1/ASK1 and p-JNK/JNK) and caspase-3/9 activation (decreased cleaved/caspase-3/9) (Figure 6B, 6C).

### JPYS reduced ovarian cell apoptosis in POF rats

The TUNEL assay was emploied to evaluate protective effects of JPYS on ovarian cell apoptosis in POF rats (Figure 6D). The number of apoptotic ovarian cells in the POF group increased compared with control group (*p* < 0.05). However, treatment with JPYS and pretreatment with triptorelin decreased the number of apoptotic ovarian cells (Figure 6D). The activity of caspase-3/9 in ovarian tissues in the POF group increased compared with control group (*p* < 0.05) but decreased after JPYS treatment and triptorelin pretreatment (Figure 6E, 6F).

### Compound analysis of JPYS-containing serum

**Figure 7:**
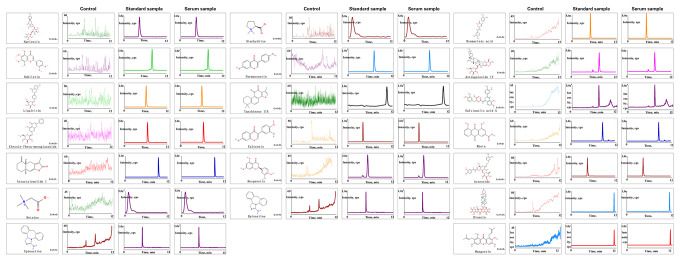
**Chemical component analysis of the JPYS-containing serum.** Preparation of JPYS-Containing Serum. Female SD rats were intragastrically administrated with normal saline (control) or JPYS (11.0 g/kg/d) once daily for seven consecutive days. Then, 17 compounds (Rhein, salvianolic acid A, liquiritin, acteoside, calycosin-7-O-β-D-glucoside, rosmarinic acid, formononetin, calycosin, astragaloside IV, atractylenolide I, dioscin, tanshinone IIA, narirutin, nobiletin, hesperetin, stachydrine, betaine) were further quantified simultaneously by HPLC-QQQ-MS/MS.

The results of compound analysis were as follows: rhein (0.127 μg/mL), salvianolic acid A (1.868 μg/mL), liquiritin (0.068 μg/mL), acteoside (0.000 μg/mL, calycosin-7-O-β-D-glucoside (0.134 μg/mL), rosmarinic acid (0.812 μg/mL), formononetin (0.010 μg/mL), calycosin (0.049 μg/mL), astragaloside IV (24.989 μg/mL), atractylenolide I (0.016 μg/mL), dioscin (1.873 μg/mL), tanshinone IIA (0.043 μg/mL), narirutin (5.145 μg/mL), nobiletin (0.064 μg/mL), hesperetin (0.467 μg/mL), stachydrine (0.015 μg/mL), and betaine (5.508 μg/mL) (Figure 7). 2D structure of chemical components were seeked in https://pubchem.ncbi.nlm.nih.gov/.

### JPYS alleviated CTX-induced apoptosis *in vitro*

**Figure 8:**
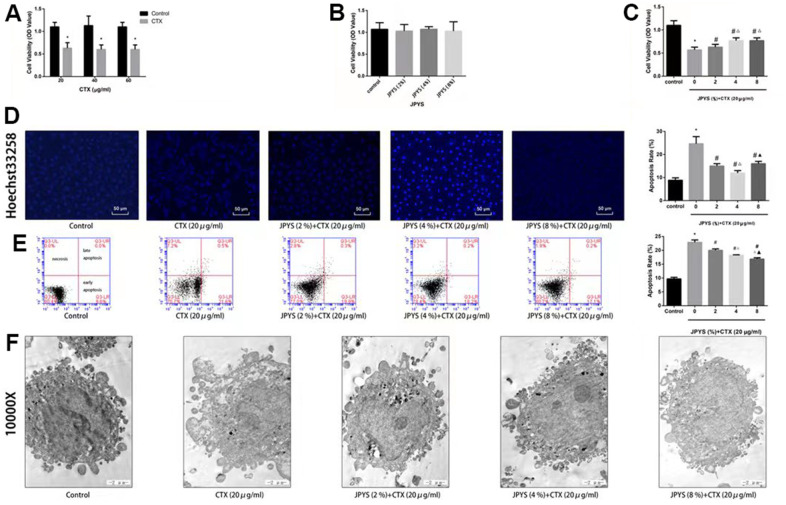
**JPYS improved decreased cell viability, increased cell apoptosis caused by cyclophosphamide (CTX) *in vitro*.** JPYS-containing serum inhibited loss of cells viability induced by CTX. (**A**) Cells were treated with CTX (20, 40, and 60 μg/ml) for 24 h; (**B**) Cells were treated with JPYS-containing serum (2, 4, 8 %); (**C**) Cells were treated with CTX (20 μg/ml) for 24 h, then treated with JPYS-containing serum (2, 4, 8 %); (**D**) Hoechst 33258 staining was used to detect the apoptosis and counted the percentage of apoptotic cells; (**E**) Cell apoptosis was measured by fow cytometry and counted the percentage of apoptotic cells: provided 2-dimensional graphical representations of PI/annexin V-FITC plots. ‘Early apoptosis’ was defifined as cells positive for annexin V-FITC only. ‘Late apoptosis’ was defifined as cells positive for annexin V-FITC and PI. ‘Necrosis’ was defifined as cells positive for PI only; (**F**) Cell apoptosis was observed by electron microscope pictures (10,000×), the scale bars represents a length of 2 μm on cells respectively. Data are shown as mean ± SD. *p < 0.05 versus control group, ^#^*p* < 0.05 versus CTX group, ^△^*p* < 0.05 versus CTX+ JPYS (2 %) group, ^▲^*p* < 0.05 versus CTX+ JPYS (4 %) group. (n=3).

To assess effects of JPYS-containing serum and CTX on cell function, a cell viability assay was emploied. Compared to control group, KGN cell viability decreased through intrevening with CTX (20, 40, and 60 μg/mL) (*p* < 0.05). The results of light microscopy revealed normal cells with long fusiform shapes in control group. Nevertheless, CTX treatment altered cell shape by shrinking cells and disrupting cell–cell interactions. Cell viability was not affected by JPYS-containing serum (2, 4, 8 %) but it was affected by pretreatment with CTX (20 μg/mL) followed by treatment with JPYS (2, 4, 8 %). At the two lowest concentrations but not at the highest concentration, JPYS-containing serum attenuated cell viability dose-dependently (*p* < 0.05) (Figure 8A–8C).

Compared to control group, CTX (20 μg/mL) induced apoptosis. At the two lowest concentrations but not at highest concentration, JPYS-containing serum attenuated the apoptosis dose-dependently (*p* < 0.05) (Figure 8D).

Compared to control group, the apoptotic rate increased in the POF group (*p* < 0.05). The apoptotic rate was lower in cells pretreated with JPYS-containing serum at concentrations of 2 and 4 % than that in untreated cells (*p* < 0.05), and the effects were dose-dependent (*p* < 0.05). However, JPYS-containing serum at its highest concentration did not protect cells from apoptosis (Figure 8E). The results of electron microscopy revealed that untreated cells were healthy, whereas CTX caused apoptosis, and JPYS-containing serum (2 and 4 %) can inhibit apoptosis induced by CTX (Figure 8F).

### JPYS improved CTX-induced mitochondrial dysfunction *in vitro*

**Figure 9:**
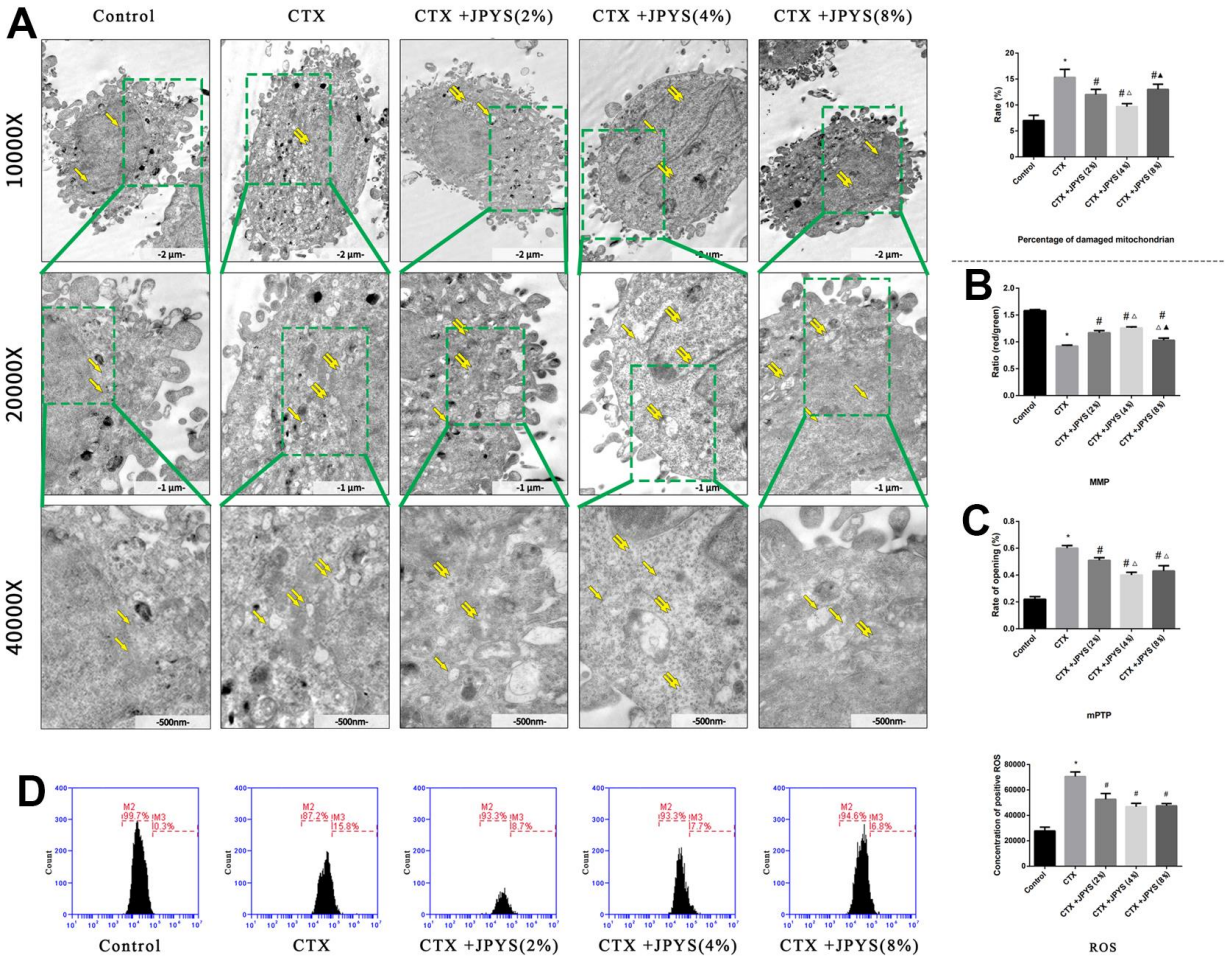
**JPYS improved mitochondrial dysfunction caused by CTX *in vitro*.** Cells were treated with CTX (20 μg/ml) for 24 h, then treated with JPYS-containing serum (2, 4, 8 %). (**A**) Electron microscope pictures (10,000×; 20,000×; 40,000×) of ovary in POF rats, the scale bars represents a length of 2 μm, 1 μm, and 500 nm on cells respectively. Abnormal mitochondrial (paired yellow arrow) morphology show that mitochondrial membrane rupture or swellings, normal mitochondrial (single yellow arrow) morphology type show that mitochondrial membrane smooth and inner carinulae distinct and percentage of damaged mitochondria; (**B**) The MMP (ratio of red/green); (**C**) The opening of the mPTP (%); (**D**) The ROS levels. Data are shown as mean ± SD. *p < 0.05 versus control group, ^#^*p* < 0.05 versus CTX group, ^△^*p* < 0.05 versus CTX+ JPYS (2 %) group, ^▲^*p* < 0.05 versus CTX+ JPYS (4 %) group. (n=3).

The percentage of damaged mitochondria was higher in the CTX group than that in the control group (*p* < 0.05). The percentage of damaged mitochondria decreased after treatment with JPYS-containing serum at concentrations of 2 or 4 % but not at a concentration of 8 % (*p* < 0.05) (Figure 9A).

Compared to control group, the MMP decreased in CTX group (*p* < 0.05). Cells pretreated with 2 or 4 %, but not 8 %, JPYS-containing serum had an increased MMP (*p* < 0.05) (Figure 9B). Compared to control group, the mPTP (%) increased in CTX group (*p* < 0.05). Cells pretreated with 2 or 4 %, but not 8 % JPYS-containing serum had a decreased mPTP (%) (*p* < 0.05) (Figure 9C). Compared to the control group, the ROS levels increased in the CTX group (*p* < 0.05), cells pretreated with 2 or 4 %, but not 8 % JPYS-containing serum had an decreased ROS levels (*p* < 0.05) (Figure 9D). As such, 4 % JPYS-containing serum was selected for subsequent experiments.

### JPYS inhibited ASK1/JNK-induced apoptosis and mitochondrial dysfunction *in vitro*

**Figure 10:**
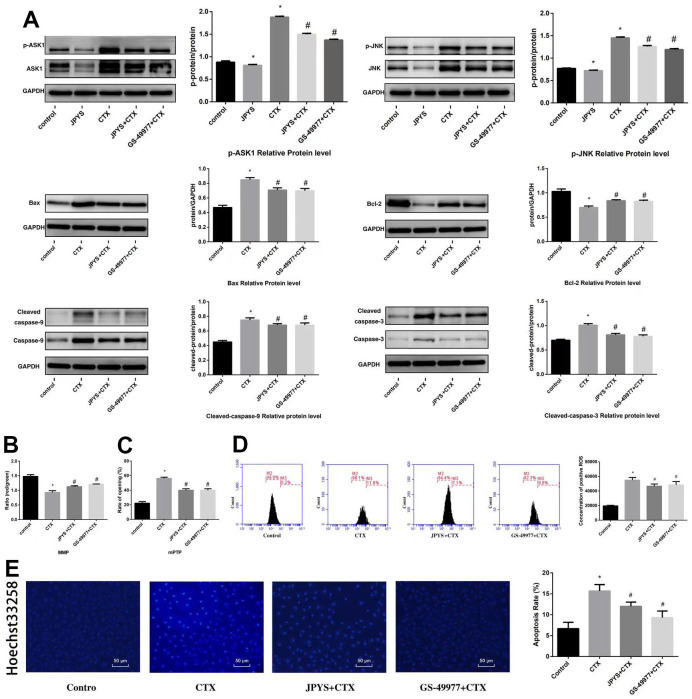
**JPYS inhibited ASK1/JNK-induced apoptosis and mitochondrial dysfunction *in vitro*.** To investigate whether the anti-apoptotic efect of JPYS-containing serum (4 %) was associated with the ASK1/JNK pathway. GS-49977 (5 μM) was the inhibitor of ASK1. (**A**) We used western blot to detect the target genes of the ASK1/JNK pathway in protein level; (**B**) The MMP (ratio of red/green); (**C**) The opening of mPTP (%); (**D**) The ROS levels; (**E**) Hoechst 33258 staining was used to detect the apoptosis and counted the percentage of apoptotic cells. Data are shown as mean ± SD. *p < 0.05 versus control group, ^#^*p* < 0.05 versus CTX group. (n=3).

To research whether anti-apoptotic effects of JPYS-containing serum were associated with ASK1/JNK pathway, GS-49977 (ASK1 inhibitor) was emploied. Compared to control group, CTX increased ASK1 and JNK phosphorylation (*p* < 0.05), whereas JPYS and GS-49977 decreased ASK1 and JNK phosphorylation (*p* < 0.05). Compared to the control group, CTX up-regulated Bax and cleaved caspase-9 levels (*p* < 0.05), whereas JPYS and GS-49977 down-regulated Bax and cleaved caspase-9 levels (*p* < 0.05). Compared to the control group, CTX decreased the Bcl-2 level (*p* < 0.05), whereas PYSP and GS-49977 increased the Bcl-2 level (*p* < 0.05) (Figure 10A). Compared to the control group, the MMP level (*p* < 0.05) was decreased but the mPTP level (*p* < 0.05) was increased in POF group. Compared with CTX group, the MMP level (*p* < 0.05) was increased but mPTP was decreased after JPYS and GS-49977 treatment (*p* < 0.05) (Figure 10B, 10C). Compared to the control group, the ROS levels increased in the CTX group (*p* < 0.05), cells pretreated with JPYS and GS-49977 had an decreased ROS levels (*p* < 0.05) (Figure 10D).

Compared to control group, CTX induced apoptosis, whereas JPYS and GS-49977 inhibited apoptosis (*p* < 0.05) (Figure 10D).

## DISCUSSION

The main findings of this *in vitro* and *in vivo* study were that granulosa cell apoptosis associated with POF and that JPYS protected ovarian tissues from damage caused by POF. As far as I am concerned, this research is firstly to study protective effects and mechanism about JPYS in POF. Mitochondrial dysfunction can induce granulosa cell apoptosis and JPYS can reduce apoptosis through mitochondrial function improved via the ASK1/JNK pathway. To investigate the damage caused by CTX and the protective mechanism of JPYS, we used CTX to build POF rats and using JPYS to research protective effects.

POF, also known as primary ovarian insufficiency (POI), affects the fertility of women of child-bearing age. The difference between menopausal and POF women is that ovaries in POF women still have a reserve. As such, it is important to understand how ovarian function can be maintained in cases of POF [19]. CTX, an alkylating chemotherapeutic drug, is one of the most damaging drugs due to its high toxicity and high risk of POF [20]. CTX can damage primordial follicles and reduce the ovarian reserve, bringing about POF and inducing cellular changes regardless of the stage of cell cycle such as genomic alterations, morphological damage, and apoptosis [21]. Many of these changes are irreversible. CTX is also used in the treatment of non-malignant diseases such as systemic lupus erythematosus [22].

Apoptosis is a type of ATP-dependent cell death induced by various extracellular and intracellular signals, and it involves several proteins and pathways such as cell death receptor as well as mitochondrial and endoplasmic reticulum pathways, with the mitochondrial pathway having the greatest role in apoptosis [23–25]. Granulosa cells produce many peptides and proteins that are related to synthesis of progesterone and estrogen. Follicular atresia, which is a key feature of POF, is caused by apoptosis of granulosa cells and oocytes [24, 26]. Thus, granulosa cell apoptosis is an initiating factor in the occurrence of POF [27].

Most traditional Chinese medicines remain uncharacterized. Astragali Radix, which was previously identified as one of the compounds in JPYS by profiling analysis [8], is used in the promotion of energy and in the improvement of hypoxia [28]. For the past two decades, it has also been widely prescribed for CKD patients. According to what I know, this study is firstly to use JPYS JPYS in the treatment of POF. At cellular level, JPYS can improve mitochondrial dynamics, promote the balance of fission/fusion, and then inhibit mitophagy and autophagy [29]. Astragali Radix is a flavonoid, and this group of compounds has been reported to improve mitochondrial function [30]. In this study, we detected the chemical components of JPYS, qualitative and quantitative analysis of 15 chemical components in decoction and qualitative and quantitative analysis of 17 chemical components in containing serum, those chemical components are very complex (Figures 1, 7).

Cyt-c release and ROS generation can induce apoptosis, and the opening of the mPTP is critical for these cellular processes. Thus, mitochondrial function is improved and apoptosis is inhibited when the opening of the mPTP is suppressed [12], and this may provide new insights into development of anti-cancer drugs such as those that target ovarian cancer [31]. The opening of mPTP, which is mediated by binding to mitochondrial inner membrane protein cyclophilin D, decreases the membrane potential, induces mitochondrial swelling, and inhibits oxidative phosphorylation. A previous study has reported that cyclosporine A binds to cyclophilin D, thereby suppressing the opening of mPTP and reducing injury [32]. Therefore, we hypothesized that JPYS has a protective function in the ovaries by improving mitochondrial function (inhibiting the opening of mPTP) in POF rats.

Rats were intraperitoneally injected with CTX (50 mg/kg) to induce granulosa cell apoptosis. The effects of JPYS on the reproductive system were assessed by measuring the levels of serum E_2_ and FSH. FSH, a pituitary hormone that binds to FSHR and activates aromatase to produce E_2_, acts on the ovaries, although E2 can inhibit the production of FSH through a negative feedback loop [33]. Both hormones are critical for follicle growth.

In POF rats, CTX affected the estrous cycle by extending the cycle length, increasing the abnormal estrous cycle rate, and decreasing the pregnancy rate, whereas opposite findings were observed after JPYS treatment, suggesting that JPYS can improve the ovarian reserve. In addition, CTX decreased the diameter of bilateral ovaries and ovarian index and ovarian function (FSH increased, E_2_ decreased, and FSH increased), whereas JPYS increased both indices. HE staining showed that CTX decreased the number of primary/secondary follicles, as well as surface area of corpora lutea, and increased the number of atretic follicles, whereas opposite findings were observed for JPYS and triptorelin, similar to Zuogui pills (ZGP) that have been previously used to treat POF in rats [34].

With regard to cell death, the number of apoptotic cells in ovarian tissues in the POF group increased. JPYS performed its anti-apoptotic effect by inhibiting expression of Bax (pro-apoptotic protein) and the activity of caspase-9/3, and increasing expression of Bcl-2 (anti-apoptotic protein) *in vivo* and *in vitro*. The decreased Bax shifted to mitochondria to integrate with increased Bcl-2, thereby forming the Bax/Bcl-2 heterodimer on the mitochondrial membrane. The Bcl-2 family is comprised of pro-apoptotic proteins (Bad, Bax, and Bid) and anti-apoptotic proteins (Bcl-xL, Bcl-2, and Bel-w) [35]. Bcl-2, a proto-oncogene located on chromosome 18a21, inhibits cancer cell apoptosis, whereas Bax is a pro-apoptotic gene located on chromosome 19q13. Both the Bax/Bcl-2 heterodimer and Bcl-2/Bcl-2 homodimer can preserve permeability of the mitochondrial membrane, and the Bax/Bax homodimer can decrease MMP. As such, the ratio of Bax and Bcl-2 in cells controls whether they will live or die [36].

The underlying mechanism of ovarian injury remains unknown, even as studies have reported the involvement of senescence and apoptosis in the loss of the ovarian reserve [37]. Mitochondrial dysfunction often associates with granulosa cell injury in POF mice [38]. The disruption of mitochondrial pathways (NF-jB/p53/PUMA and PI3K/Akt/Bad) can cause mitochondrial dysfunction [18, 39, 40]. In this study, CTX caused mitochondrial swelling and membrane rupturing, and increased the number of damaged mitochondria, whereas JPYS had a protective effect *in vivo* and *vitro* (Figures 4A, 9A).

There is an association between the disruption in mitochondrial homeostasis and the pathophysiology of POF [41], as impaired mitochondrial function and increased oxidative stress are key aspects of POF [38]. JPYS decoction can decrease the chronic kidney disease (CKD)-induced imblance of mitochondrial quality control processes, via increasing mitochondrial biogenesis, renewing the balance between fusion and fission, and decreasing autophagy-lysosome pathway (mitophagy) in past research [29].

We established and used the POF rat model to examine mitochondrial function. RCR, the MMP, oxygen consumption rate, mitochondrial respiratory chain complex enzymes and ATP were reduced in POF group, whereas ROS production and mPTP opening (%) were increased in the POF group. JPYS treatment improved the mitochondrial function indices *in vitro* and *in vivo*. Others have used the copy number of mtDNA to evaluate the number of mitochondria [13, 42]. Although the mechanism of mtDNA repair is unknown, mtDNA is near the respiratory chain, so mtDNA is more vulnerable when exposed to oxidative stress. We calculated ratio of long/short fragments as an indicator of mtDNA integrity, and the ratio of long/short fragments was decreased in the POF group, but increased in the JPYS treatment group.

Mitochondrial biogenesis involves many genes such as OPA1, Mfn1, and Mfn2. As a regulatory gene, OPA1 regulates mitochondrial dynamics. L-OPA1 overexpression can reduce neuronal apoptosis through increasing Bcl-2 level and decreasing Bax level, as well as caspase-3 activation, so those genens are related with apoptosis. Furthermore, L-OPA1 overexpression can modulate mitochondrial dysfunction by reversing mitochondrial damage, reducing oxidative stress and energy deficits, preserving mitochondrial integrity, and promoting mitochondrial biogenesis in the brain [43]. Other proteins involved in mitochondrial dynamics are Drp1 and Mfn [44]. Mfn2, a conserved dynamin-like GTPase situated on outer membrane of mitochondria, affects mitochondrial structure and function by modulating fission and fusion [45]. Many studies have demonstrated that Mfn2 can control the respiratory chain, MMP, metabolic processes, and apoptosis [46]. Furthermore, Mfn2 performs a critical function in preserving the integrity of mtDNA [47]. In our research, OPA1 and Mfn1/2 mRNA and protein levels decreased in the POF group, whereas JPYS treatment and triptorelin pretreatment increased these mRNA and protein levels. It also decreased the ratio of long/short fragments, consistent with results of a previous study [47]. Mitochondria undergo fission and fusion, and these dynamic processes are critical for the maintenance of mitochondrial size, shape, and organization. PGC-1α, an important transcriptional co-activator, can modulate key factors, including Nrf1 and Tfam, and they are considered to up-regulate mitochondrial biogenesis [48]. Drp1, a major regulatory factor of mitochondrial fission. Drp1 link to mitochondria increases apoptosis [49]. Fis1 can induce apoptosis by interacting with endoplasmic reticulum-localized Bap31 in Bax/Bak-mediated permeabilization in mitochondrial outer membrane, resulting in cyt-c release even cell death [50]. Fis1 has been be closely related with reducing GTPase activity of Mfn1/2 and OPA1 [51]. Our results demonstrated that the levels of Drp1 and Fis1 increased, yet PGC-1α level decreased in POF group, showing an imbalance of fision and fusion in mitochondria. As the two main pathways (extrinsic death receptor and intrinsic mitochondrial pathways) of the regulating cell apoptosis, mitochondrial apoptosis pathway control the mPTP (relese the ROS and Cty-c) to induce apoptosis. Furthermore, mitochondrial dynamics can regulate the cell apoptosis, fission and fusion are process of mutual cooperation which can modulate the morphology and the number of mitochondria, this process determine mitochondrial mass (removing poor quality), and mitochondrial mass determine cell apoptosis.

ASK1 is a ubiquitously expressed MAP3K that is activated by several stimuli, as well as overexpressed in neurodegenerative disorders, cancer, and infammatory diseases [52]. Activated ASK1 activates downstream kinases, such as JNK and p38, leading to infammatory cytokine expression and apoptosis [53]. The ASK1 inhibitor selonsertib (GS-49977) is a latent therapeutic medication for early curing ALF, in that it reduces JNK-mediated Drp1 translocation in mitochondria and then alleviated mitochondrial injury [54]. The ASK1/JNK signaling pathway exerts a critical role in the initiation of mitochondria-mediated apoptosis, ASK1/JNK pathway can improve the MMP and control the opening of mPTP (Figure 6) [55]. Our results showed that JPYS affected the expression of ASK1/JNK pathway-related proteins, inhibited ASK1 and JNK phosphorylation, and decreased Cyt-c and cleaved caspase-3/9 expression. GS-49977 elicited similar effects *in vitro* (Figure 10). Taken collectively, these results showed that JPYS can improve mitochondrial function by decreasing injury and increasing mitochondrial function, and inhibiting CTX-induced apoptosis by inhibiting the ASK1/JNK pathway.

## CONCLUSIONS

In this study, we isolated mitochondria and showed that CTX damaged mtDNA, disrupted mitochondrial respiratory function, and produced ROS, thereby causing ovarian injury. Mitochondrial swelling was led by the opening of mPTP in POF rats. The opening of mPTP can induce the flow back of protons from the mitochondrial membrane to the matrix, and then decreasing ATP synthesis and the MMP and leading to metabolic abnormalities. However, JPYS inhibited apoptosis through regulating mitochondrial function via the inhibition of the ASK1/JNK pathway in POF rats. We considered that JPYS may be emploied as a potential therapeutic medication for POF, and mitochondria can be seen as a potential therapeutic target. However, mechanism research is not deep enough and we did not employ the bioinformatics methods, those will be performed in future.
