# Correction for: Jian-Pi-Yi-Shen decoction inhibits mitochondria-dependent granulosa cell apoptosis in a rat model of POF

**DOI:** 10.18632/aging.204637

**Published:** 2023-03-30

**Authors:** Xiao-Lin Jiang, He Tai, Jin-Song Kuang, Jing-Yi Zhang, Shi-Chao Cui, Yu-Xuan Lu, Shu-Bo Qi, Shi-Yu Zhang, Shun-Min Li, Jian-Ping Chen, Xian-Sheng Meng

**Affiliations:** 1 Department of Nephrology, The Fourth of Affiliated Hospital of Guangzhou University of Traditional Chinese Medicine (Shenzhen Traditional Chinese Medicine Hospital), Guangzhou University of Traditional Chinese Medicine, Shenzhen, China; 2 Key Laboratory of Ministry of Education for Traditional Chinese Medicine Viscera-State Theory and Applications, Liaoning University of Traditional Chinese Medicine, Shenyang, China; 3 College of Pharmacy, Liaoning University of Traditional Chinese Medicine, Dalian, China; 4 Department of Internal Medicine, Liaoning Provincial Corps Hospital of Chinese People’s Armed Police Forces, Shenyang, China; 5 Department of Endocrinology and Metabolism, The Fourth People’s Hospital of Shenyang, Shenyang, China; 6 Department of Pharmacy, General Hospital of Northern Theater Command, Shenyang, China; 7 NHC Key Laboratory of Male Reproduction and Genetics, Guangdong Provincial Reproductive Science Institute (Guangdong Provincial Fertility Hospital), Guangzhou, China; 8 College of Basic Medical Science, Chinese Capital Medical University, Beijing, China

**Keywords:** Jian-Pi-Yi-Shen, premature ovarian failure, mitochondrial dysfunction, granulosa cell, apoptosis

**This article has been corrected:** The authors corrected **Table 3, “Sequence of primers for RT-PCR and long PCR,”** because they forgot to update this table before submission. The primers used in the study were changed three times before a set that worked was synthesized and verified by “Sangon Biotech.” They also found and corrected a duplication in **Figure 5B** created during the figure assembly - column chart “PGC-1&alpha;” replicates column chart “Fis1.” Correction was done with data from the original sets of Western blots for PGC-1&alpha; protein. These corrections have no impact on the experimental outcome or conclusions.

The corrected **Table 3** and **Figure 5** are presented below.

**Table:** 

Table 3. Sequences of primers for RT-PCR and long PCR.


**Table:** 

Target Gene	Primer Sequence	Tm (°C)
OPA1	Forward: 5’-TGGTTCGAGAGTCGGTTGAA-3’	56
	Reverse: 5’- CCTCCCAGTGCTTTGGAGTA -3’	56
Mfn1	Forward: 5’-GGGAAGACCAAATCGACAGA-3’	57
	Reverse: 5’-CAAAACAGACAGGCGACAAA-3’	57
Mfn2	Forward: 5’-GAGAGGCGATTTGAGGAGTG-3’	58
	Reverse: 5’-CTCTTCCCGCATTTCAAGAC-3’	56
Drp1	Forward: 5’-GCCCGTGGATGATAAAAGTG-3’	56
	Reverse: 5’-TGGCGGTCAAGATGTCAATA-3’	56
Fis1	Forward: 5’-AGATGGACTGGTAGGCATGG-3’	56
	Reverse: 5’-GACACAGCCAGTCCAATGAG-3’	56
PGC-1α	Forward: 5’-GGACGAATACCGCAGAGAGT-3’	59
	Reverse: 5’-CCATCATCCCGCAGATTTAC-3’	56
Tfam	Forward: 5’-TCACCTCAAGGGAAATTGAAG-3’	55
	Reverse: 5’-CCCAATCCCAATGACAACTC-3’	56
Long Fragment	Forward:5’-AAAATCCCCGCAAACAATGACCACCC-3’	72
	Reverse: 5’-GGCAATTAAGAGTGGGATGGAGCCAA-3’	72
Short Fragment	Forward: 5’-CCTCCCATTCATTATCGCCGCCCTGC-3’	60
	Reverse: 5’-GTCTGGGTCTCCTAGTAGGTCTGGGAA-3’	60
Bax	Forward: 5’-GCGATGAACTGGACAACAAC-3’	57
	Reverse: 5’-GATCAGCTCGGGCACTTTAG-3’	58
Bcl-2	Forward: 5’-CGAGTGGGATACTGGAGATGA-3’	58
	Reverse: 5’- GACGGTAGCGACGAGAGAAG-3’	59
Caspase-3	Forward: 5’-GACTGGAAAGCCGAAACTCT-3’	55
	Reverse: 5’-TGCCATATCATCGTCAGTTCC-3’	54
Caspase-9	Forward: 5’-CAGAGGTTCTCACACCAGAAA-3’	54
	Reverse: 5’-TGCCATATCTGCATGTCTCTC-3’	54
ASK1	Forward: 5’-GACAAGAGAGCCTGTGCTAAT-3’	54
	Reverse: 5’-TCTCCGTGCAACCACATAC-3’	55
JNK	Forward: 5’-GGATTTGGAGGAGCGAACTAA -3’	54
	Reverse: 5’-CATTGACAGACGGCGAAGA-3’	55
Cty-c	Forward: 5’-GGACAGCCCCGATTTAAGTA-3’	57
	Reverse: 5’-TCAATAGGTTTGAGGCGACAC-3’	58
GAPDH	Forward: 5’- AGGTCGGTGTGAACGGATTTG -3’	58
	Reverse: 5’- GGGGTCGTTGATGGCAACA-3’	58

**Figure 5:**
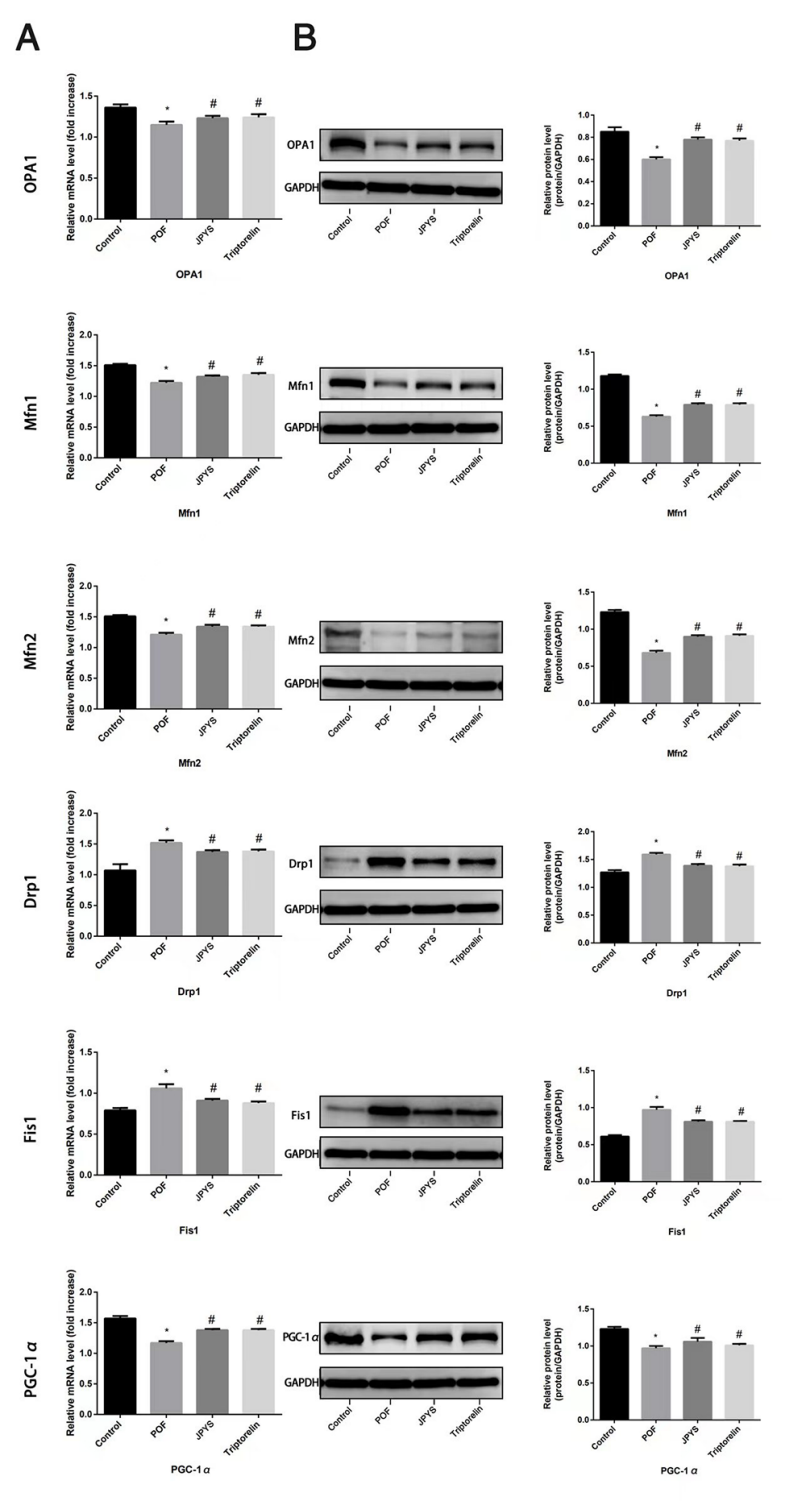
**JPYS improved mitochondrial biogenesis and dynamics in premature ovarian failure (POF) rats.** Rats were treated with JPYS (11.0 g/kg.d) and pre-treated with triptorelin (1.5 mg/kg) followed by intraperitoneally injected cyclophosphamide (50 mg/kg). We used real-time qPCR and western blot to detect mitochondrial biogenesis and dynamics. We chose OPA1, Mfn1, and Mfn2 to represent mitochondrial biogenesis function, and PGC-1α to represent the dynamic mitochondrial fusion, and Drp1 and Fis1 to represent mitochondrial ﬁssion. The expression of OPA1, Mfn1, Mfn2, PGC-1α, Drp1, and Fis1 in mRNA (**A**) and protein (**B**) levels. Data are shown as mean ± SD. *p < 0.05 versus control group, ^#^*p* < 0.05 versus POF group, ^△^*p* < 0.05 versus JPYS group. (n=6).

